# Evaluation of miR-148a-3p and miR-106a-5p as Biomarkers for Prostate Cancer: Pilot Study

**DOI:** 10.3390/genes15050584

**Published:** 2024-05-04

**Authors:** Roxana Andra Coman, Vlad Horia Schitcu, Liviuta Budisan, Lajos Raduly, Cornelia Braicu, Bogdan Petrut, Ioan Coman, Ioana Berindan-Neagoe, Nadim Al Hajjar

**Affiliations:** 1 Department of Urology, “Iuliu Hatieganu” University of Medicine and Pharmacy, 400012 Cluj-Napoca, Romania; dr.roxanacoman@yahoo.com (R.A.C.); bogdan.petrut@gmail.com (B.P.); jcoman@yahoo.ro (I.C.); 2 Department of Urology, “Prof Dr. Ion Chiricuta” Oncology Institute, 400015 Cluj-Napoca, Romania; schitcu@yahoo.com; 3 Research Center for Functional Genomics, Biomedicine and Translational Medicine, “Iuliu Hatieganu” University of Medicine and Pharmacy, 400337 Cluj-Napoca, Romania; liviuta.petrisor@umfcluj.ro (L.B.); lajos.raduly@umfcluj.ro (L.R.); cornelia.braicu@umfcluj.ro (C.B.); 4 Department of Surgery, “Iuliu Hatieganu” University of Medicine and Pharmacy, 400012 Cluj-Napoca, Romania; nadim.alhajjar@umfcluj.ro; 5 Department of Surgery, Regional Institute of Gastroenterology and Hepatology “Octavian Fodor”, 400394 Cluj-Napoca, Romania

**Keywords:** microRNAs, miR-106a-5p, miR-148a-3p, prostate cancer

## Abstract

MicroRNAs (miRNAs) are a class of small non-coding RNAs that may function as tumor suppressors or oncogenes. Alteration of their expression levels has been linked to a range of human malignancies, including cancer. The objective of this investigation is to assess the relative expression levels of certain miRNAs to distinguish between prostate cancer (PCa) from benign prostatic hyperplasia (BPH). Blood plasma was collected from 66 patients diagnosed with BPH and 58 patients with PCa. Real-time PCR technology was used to evaluate the relative expression among the two groups for miR-106a-5p and miR-148a-3p. The significant downregulation of both miRNAs in plasma from PCa versus BPH patients suggests their potential utility as diagnostic biomarkers for distinguishing between these conditions. The concurrent utilization of these two miRNAs slightly enhanced the sensitivity for discrimination among the two analyzed groups, as shown in ROC curve analysis. Further validation of these miRNAs in larger patient cohorts and across different stages of PCa may strengthen their candidacy as clinically relevant biomarkers for diagnosis and prognosis.

## 1. Introduction

Prostate cancer (PCa) is the second most prevalent cancer among men globally, with higher incidence and mortality in Northern and Western Europe, the Americas, Australia, and the Caribbean. The increasing prevalence of screening is expected to decrease mortality rates, while it may lead to overdiagnosis [1]. The conclusive diagnosis of PCa is determined by the histopathological result after analysis of the prostate biopsy cores.

Prostate biopsy is indicated when the levels of the prostate-specific antigen (PSA) are elevated beyond normal ranges or when there are other clinical indications suggestive of prostate cancer, such as abnormalities found during a digital rectal examination (DRE) or suspicious findings on imaging studies like Magnetic Resonance Imaging (MRI) or ultrasound. The PSA is not a tumor-specific marker but rather an organ-specific marker, which is why we can find PCa at a normal value of PSA or elevated PSA levels in other conditions, most commonly benign prostatic hyperplasia (BPH) [2]. A suspicious DRE associated with normal PSA (≤4 ng/mL) has a positive predictive value of 5–30% [3], but the association between suspicious DRE and elevated PSA can double the chance of finding a positive prostate biopsy (48%) [4]. There has been an increasing use of MRI in various aspects of PCa management in recent years. The average positive predictive value is correlated with the PI-RADSv2.1 score. The PIRADSv2.1 3 score was 16% and 59% and 85% for PIRADSv2.1 scores 4 and 5 [5]. Such clinical diagnosis leads to tissue biopsy, which is the only way to differentiate between prostate diseases. Many useless biopsies are performed, exposing patients to risks and stress. It is necessary to develop novel biomarkers that possess high sensitivity and specificity to address these diagnostic procedures. It is also essential to discriminate between indolent and aggressive tumors, particularly in the early stages of the disease. On the other hand, PCa can be found incidentally after surgery for BPH. The rate of incidental PCa ranges from 5.64% to 23.3% [6,7]. However, there are surgical techniques that vaporize the prostatic tissue, and there is no histopathological evaluation such as Green Laser, Thulium vaporization, or Rezum. For all these reasons, having an accurate diagnosis before recommending surgical options is important.

MicroRNAs (miRNAs) are defined as a group of small non-coding RNA transcripts, typically consisting of around 22 nucleotides in length. They have the potential to act as both tumor suppressors and oncogenes, as miRNA alteration of the expression levels has been related to diverse forms of human malignancies. These transcripts are investigated to sustain the diagnosis and treatment of cancer, including PCa [8]. They bind to matching sequences in the 3′UTR of target miRNAs using a conserved ‘seed sequence’ area, inhibiting the target mRNAs’ translation [9].

Additionally, miRNAs are abundantly and stably expressed in various biological contexts and can be readily detected in tissues and biofluids, such as plasma. This characteristic makes them promising candidates for biomarker discovery and noninvasive diagnostic applications in various diseases, including cancer. Their remarkable stability also makes them promising candidates for noninvasive biomarkers in cancer diagnosis and prognosis [10,11].

Different types of cancer, including PCa, have been shown to have different miRNA expression patterns between tumor and normal tissues, plasma, and exosomes [12,13]. In PCa, various studies have demonstrated that miRNAs regulate numerous cellular and molecular mechanisms. Some miRNAs have been identified as upregulated and others as downregulated in PCa [14]. The selection of miR-106a-5p and miR-148a-3p for analysis in PCa is motivated by their potential as biomarkers, their functional significance in cancer biology, and their implications for PCa treatment strategies. Studying these miRNAs’ expression profiles and regulatory functions in PCa may contribute to a better comprehension of the disease and the progress of improved diagnostic and therapeutic approaches. miR-106a-5p was identified to be downregulated in the plasma samples of PCa patients compared to those of BPH patients [15] and is upregulated in metastatic PCa [16]. MiR-148a-3p is downregulated when comparing 0PCa patients with healthy controls [17] and metastatic PCa [18]. MiR-106a-5p and miR-148a-3p are still under research as cancer biomarkers.

This study aimed to evaluate the relative expression levels of miR-106a-5p and miR-148a-3p, leading to possible implications as biomarkers in the differentiation of PCa from BPH when analyzed in the blood plasma of patients using real-time PCR technology.

## 2. Materials and Methods

### 2.1. Samples Collection of Prostate Adenoma and Prostate Cancer Patients

From July 2020 to March 2023, 124 patients were prospectively enrolled, of which 66 patients were diagnosed with BPH and 58 with PCa. All patients participating in the present investigation provided informed consent by the University Ethics Committee. The current investigation received approval from the University of Medicine and Pharmacy ‘Iuliu Hatieganu’ (UMPhIH) ’s ethical committee under reference numbers 263/23.07.2020 and 294/01.09.2021. The exclusion criteria for both groups were no other malignancy, and for the BPH group, also a PSA level > 4 ng/mL. The PCa group included 58 patients who endured laparoscopic radical prostatectomy. The BPH group included 66 patients who underwent Thulium vapor resection or transurethral resection of the prostate (TUR-P) and were diagnosed with BPH. All patients underwent fasting blood sampling. This classification categorizes PCa patients based on specific criteria. Patients with a prostate-specific antigen (PSA) level below 10 ng/mL and a Gleason score (GS) below 7, along with a clinical stage of cT1c or cT2a, are considered low risk. Patients with a PSA level between 10 and 20 ng/mL, a GS of 7, or a clinical stage of cT2b are classified as intermediate risk. Patients with a PSA level ≥ 20 ng/mL, a GS above 7, or a clinical stage of cT2c are classified as high risk. The relative expression levels of selected serum miRNAs were assessed in the PCa and BPH groups using a TaqMan qRT-PCR approach, which is considered the golden standard for miRNA expression evaluation.

### 2.2. RNA Isolation and Extraction

RNA was extracted from 124 patients’ plasma with Plasma/Serum RNA Purification Kits (Norgen Biotek Corp, Thorold, ON, Canada) according to the manufacturer’s procedure. The concentration of RNA was evaluated by the NanoDrop2000 series spectrophotometer (Thermo Fischer Scientific, Waltham, MA, USA), and the samples were diluted to a final concentration of 50 ng/μL for all the samples used in the study.

### 2.3. cDNA Synthesis and qRT-PCR for miR-106a-5p and miR-148a-3p

miRNA expression levels were detected in 124 samples. After quantification, 50 ng of total RNA was reverse-transcribed into cDNA using a TaqMan MicroRNA Transcription kit (Thermo Fischer Scientific, Waltham, MA, USA) and specific TaqMan microRNA primer assay (Thermo Fischer Scientific, Waltham, MA, USA) for the selected miRNAs (miR-106a-5p and miR-148a-3p) respecting the manufacturer’s protocol. The primer sequences for miRNAs are included in Table 1.

qRT-PCR reaction was carried out in a volume of 10 μL using 5 μL of cDNA (diluted 1:4 with nuclease-free water), 5.03 μL TaqMan Fast Advanced Master MIX (Applied Biosystems, Foster City, CA, USA), and 0.47 μL primer for each miRNA in ViiA7 (Applied Biosystems, USA) PCR machine. The reactions were set up as follows: the initial denaturation step at 50 °C for 2 min and 95 °C for 2 s, followed by 40 cycles of 95 °C for 1 s, and 60 °C for 20 s. RNU48 and U6 were used as housekeeping miRNAs. ΔΔct method was used to analyze the obtained CT values [19]. The qRT-PCR was duplicated for all the tested samples, and the outliers were removed from the analysis.

### 2.4. Biological Relevance of hsa-miR-106a-5p and hsa-miR-148a-3p

*DIANA*-*miRPath* was used to perform miRNA pathway analysis (KEGG) for the selected two transcripts [20]. DIANA-miRPath, a web server for miRNA pathway analysis, uses the predicted miRNA targets generated by the DIANA-microT-CDS algorithm. These algorithms predict target genes based on sequence complementarity between miRNAs and the 3′ untranslated regions (UTRs) of mRNA transcripts. By default, DIANA-miRPath combines all miRNA target genes and calculates *p* values to assess the KEGG pathway enrichment of these target genes using bioinformatics tools such as TarBase v7.0.

Then, Minturnet, linked to MiRTarBase, was used to explore and analyze miRNA–gene regulatory networks. This is a valuable resource for understanding the post-transcriptional regulation mediated by miRNAs and its implications in various biological processes and diseases. It incorporates only interactions with strong experimental validation, such as luciferase assay and Western blotting [21].

### 2.5. Statistical Analysis

Statistical analysis was executed with GraphPad Prism software.9 (GraphPad Software, San Diego, CA, USA) using the *t*-test method. *p*-value < 0.05 was considered to indicate a statistically significant difference among the relative expression levels of the analyzed transcripts. The analyzed transcripts’ specificity and sensitivity as biomarkers were combined in the receiver operating characteristic (ROC) curve analysis and reported in areas under the curve (AUCs). The combined ROC curve for the two analyzed transcripts was created using the CombiROC online tool [22].

## 3. Results

### Patients’ Characteristics and Expression of Candidate miRNAs

Out of the 124 patients included in the present investigation, 58 were diagnosed with PCa and 66 with BPH. The mean age of the BPH group was 66.65 years (range: 40–91), and the mean PSA was 2.52 ng/mL (range: 0.15–3.92). The clinical and histopathologic data of the PCa patients are summarized in Table 2.

**Figure 1:**
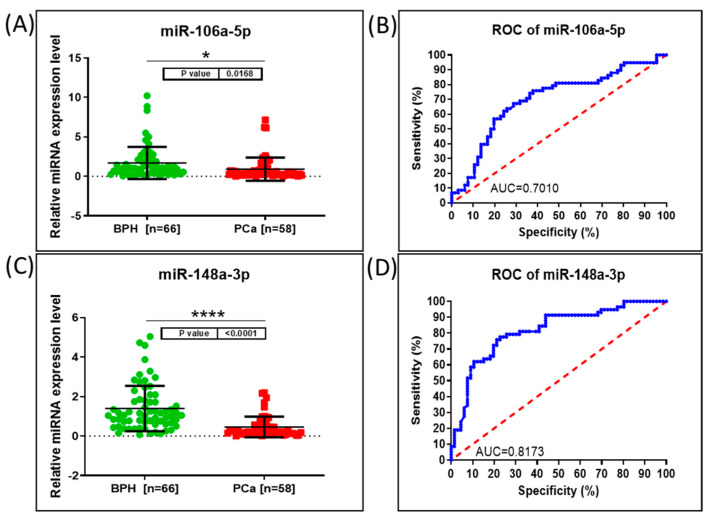
qRT-PCR evaluation of the relative expression levels of miR-106a-5p and miR-146a-3p in plasma BHP and PCa patients. (**A**) Graphical representation of the relative expression levels calculated using ΔΔct method for miR-106a-5p in plasma PCa patients versus BPH plasma, mean ± SD (Standard Deviation). The data were normalized using U6 and RNU48 (* *p* = 0.0168). (**B**) ROC curve for miR-106a-5p, displaying the specificity and sensitivity for the discernment among relative expression levels in plasma for PCa patients versus BPH, AUC = 0.7010. (**C**) Graphical representation of the expression levels calculated using ΔΔct method for miR-146a-3p in plasma PCa patients versus BPH, mean ± SD. (**D**) ROC curve for miR-148a-3p, AUC = 0.8173. The data were normalized using U6 and RNU48 (**** *p*-value < 0.0001). Abbreviations: ROC, receiver-operating characteristic; AUC, area under ROC curve.

**Figure 2:**
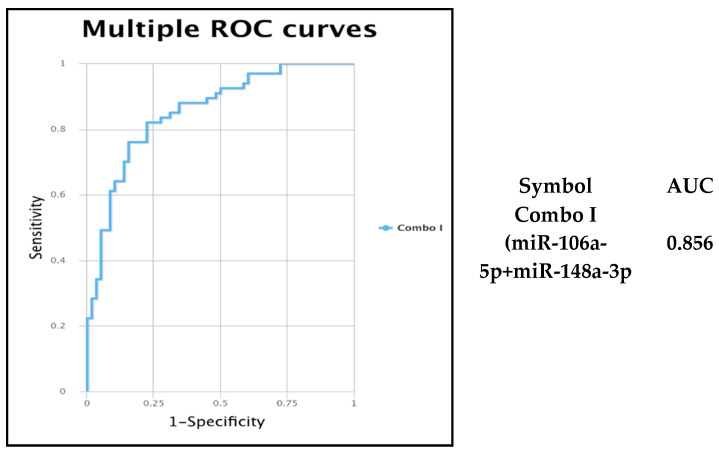
ROC curve for miR-106a-5p+miR-148a-3p, exhibiting the specificity and sensitivity for the discrimination among expression levels of these transcripts among the two analyzed groups, AUC = 0.856. Abbreviations: ROC, receiver-operating characteristic; AUC, area under ROC curve.

Both miRNAs revealed alterations in relative expression, and the transcripts proved to be downregulated in the PCa group compared to in the BPH group (Figure 1). Mir-148a-3p exhibited a highly significant modification with a *p*-value < 0.0001, while mir-106a-5p had a *p*-value of 0.0168 when comparing the PCa group versus the BHP group. These findings suggest that these miRNAs may play essential roles in distinguishing between these two groups and could potentially serve as biomarkers for characterizing differences in their molecular profiles. We performed a receiver operating characteristic curve (ROC) analysis of the relative expression levels of miR-106a-5p and miR-148a-3p in PCa patient plasma versus BPH plasma, and we calculated the area under the ROC curve (AUC) (Figure 1). Both miR-106a-5p and miR-148a-3p demonstrated significant sensitivity and specificity in differentiating the PCa versus BPH groups. Respectively, the AUC of diagnosis in the combined analysis was superior to that of individual cases. Thus, using the two transcripts was more specific to predicting PCa diagnosis and demonstrated higher specificity and sensitivity than the single-transcript evaluation (Figure 2).

**Figure 3:**
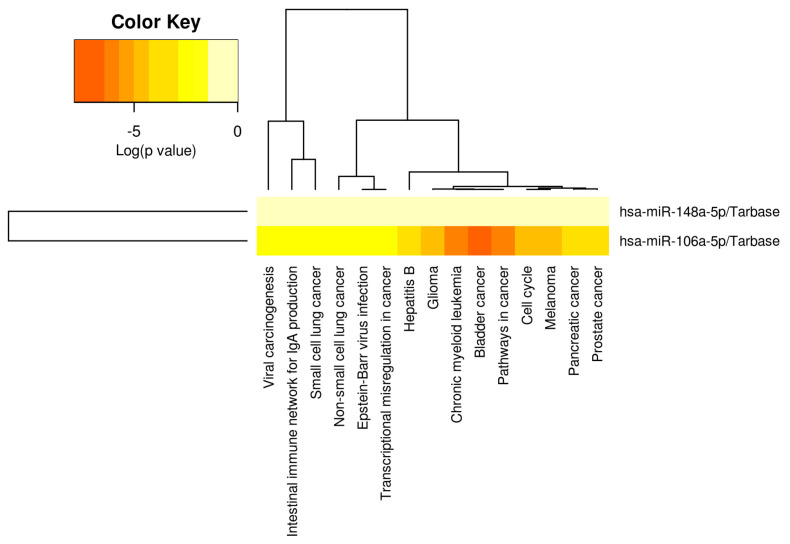
Heatmap of KEGG pathways, generating the enrichment analysis for the two miRNA target genes using DIANA-mirPath. miR-148a-5p and miR-106a-5p were proven to be connected with multiple pathways, with a particular interaction with cancer-specific signaling (DIANA-mirpath computes log_10_ *p*-values). The color legend is in the upper left corner red corresponds to a higher statistical significance, while yellow signifies a lower statistical significance (*p* < 0.05).

**KEGG pathway analysis of the two miRNA signature target genes.** The miRNAs chosen in our investigation have been linked to various cancers and diseases in prior research (Figure 3). Nonetheless, we examined whether the target genes of these two transcripts showed enrichment in KEGG pathways to unveil their biological relevance. KEGG pathway analysis of miR-106a-5p and miR-148a-3p gains insights into their potential roles in cellular pathways and processes like cell cycle regulation or transcriptional misregulation in cancer. This analysis will contribute to comprehending the selected miRNAs’ biological significance in cancer biology.

**Figure 4:**
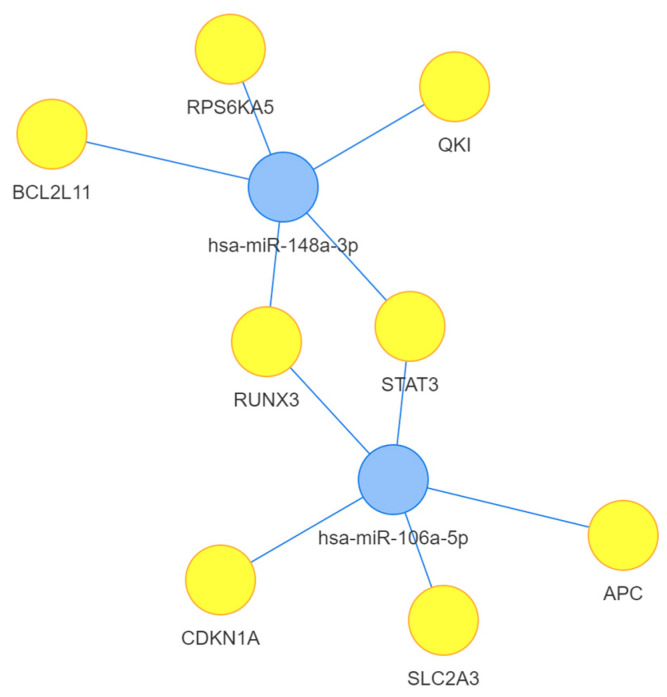
miRNA–mRNA interaction network generated using the Mienturnet online interface database, selecting only robust experimental evidence (from data generated using luciferase assay and Western blotting). This visualization will help to understand the regulatory relations between miRNAs and their target genes. Blue circles are used to present the miRNAs; yellow circles are the target genes of our selected transcripts [23].

**Network microRNA–gene interaction**. A network miRNA–gene interaction was generated using a Mienturnet online interface (linked to miRTarBase); in this case, we only selected robust experimental validation (e.g., luciferase assay, Western blotting) for the target genes (Figure 4). This short-target gene list is based on experimentally validated miRNA–target gene interactions from databases such as miRTarBase; the interaction was filtered through robust experimental evidence, such as luciferase assay or Western blotting validation. This network analysis provides valuable insights into the regulatory roles of these transcripts and their experimentally validated target genes.

## 4. Discussion

PCa ranks as the second most frequently diagnosed cancer in men and is linked to a notable number of cancer-related fatalities [1]. Therefore, early diagnosis is essential for successful treatment. Today, serum PSA is the marker used to diagnose PCa, but serum PSA levels can be elevated in other conditions, such as BPH or prostatitis, affecting its specificity [2]. Also, despite following the recommendations, we may obtain a negative prostate biopsy. In addition, the PSA level correlates poorly with tumor aggressiveness, making it an inadequate predictor of disease evolution. For all these situations, we need new biomarkers for the easy and accurate diagnosis and prognosis of PCa [24]. Several studies demonstrated that miRNA dysregulation can be a potential diagnostic modality for PCa [25]. Also, the association of TP53 with miRNAs can play a relevant role in controlling a tumor as a potential therapeutic target and biomarker in PCa [26].

This study aims to characterize the impact of miR-106a-5p and miR-146a-3p in diagnosing patients with PCa by comparing plasma from patients diagnosed with BPH or PCa. Our study found that miR-106a-5p and miR-146a-3p were significantly downregulated in the plasma samples of PCa patients versus BPH patients.

This KEGG analysis sustains the rationale for proposing miR-106a-5p and miR-146a-3p as biomarkers, considering their multiple known roles in cellular processes and the specific discrimination between PCa patients and BPH with a high sensitivity, as we can observe from ROC curves.

Cochetii et al. reported that miR-106a-5p was downregulated in the serum of PCa patients compared to in that of BPH patients, and it was correlated with increased malignancy. It considerably decreased in high-risk patients, allowing for differentiation between pGS6 and pGS7 from pGS8 [15]. Mir-106a-5p may be considered an essential target for treating metastatic PCa. Apelin, an endogenous peptide implicated in the progression of multiple cancers, including lung, hepatocellular, and colon cancer [27], facilitates TIMP-2-dependent PCa cell migration and invasion. The overexpression of miR 106-a-5p mediates the promotion of PCa motility induced by apelin via the c-Src/PI3K/Akt signaling cascades. Inhibition of apelin has been found to reduce PCa metastasis in an orthotopic mouse model. These findings suggest that apelin could be a novel therapeutic target in metastatic PCa [28]. Dhar et al. showed that miR-106a-5p expression is significantly associated with PCa progression and validated a PTEN tumor suppressor as a critical target of this oncomiR in PCa cells with a possibility of being used as a chemopreventive and predictive biomarker in the development of PCa [29]. Yang et al. also support the implications of miR-106a-5p in the therapy of PCa through the long non-coding RNAs MAGI2-AS3. The expression pattern showed miR-106a-5p to be upregulated in hormone-sensitive and castration-resistant PCa. MAGI2-AS3 was abnormally decreased in castration-resistant PCa and negatively associated with GS and lymph node involvement. Decreased MAGI2-AS3 could serve as a predictor of poor prognosis in PCa. MAGI2-AS3 is primarily cytoplasmic and inhibits the initiation and progression of PCa by mediating the expression of RAB31 through miR-106a-5p [16]. This finding underscores the importance of exploring miRNAs as potential targets for developing novel treatment strategies for PCa.

The dysregulation of miR-148a-5p has been observed across various types of cancer, such as colorectal, gastric, and hepatocellular carcinoma. Studies have shown that miR-148a-5p is a tumor suppressor by targeting oncogenes or genes in tumor progression pathways [30]. Therefore, the decreased expression of miR-148a-5p in cancer tissues versus normal tissues suggests its potential as a diagnostic biomarker for cancer.

Dybos et al. have identified miR-148a-3p as a promising diagnostic marker for PCa. Mir-148a-3p was upregulated in the serum of patients with PCa compared to that of healthy controls. It was also detected in prostate tissue; however, distinguishing it was challenging due to the heterogeneity of prostate tissue. It appears that miR-148a-3p is present in both BPH and pGS4 [17]. Another study reported that the expression of miR-148a-3p was increased in both tissue and plasma samples compared with those of healthy controls [31].

Moreover, miR-148a-3p can be used for PCa diagnosis, cancer stage assessment, and postoperative recurrence prediction in PCa patients. According to He W et al., miR-148a-3p can discriminate between GS < 7 and GS ≥ 7 cases. However, preoperative serum levels of miR-148a-3p in combination with miR-485-5p provide a much improved prediction [32].

Elevated levels of miR-148a-3p have been observed in the serum and urine of PCa patients compared to those of healthy controls [17,33] in prostate tumor tissue compared to in adjacent prostatic normal tissue [34]. In contrast, miR-148a-3p levels have been reported to be lower in CRPC cell lines PC3 and DU145 compared to in lines that represent a therapy-responsive model for the study of this disease [35]. Likewise, in PCa patients, the downregulation of miRNAs has been observed in CRPC versus BPH cases and in high-grade versus low-grade tumors [18,36]. Also, there have been reports of a decrease in the expression of miR-148a-3p in patients at a high risk of experiencing biochemical failure [37]. The value of the downregulated expression of miR-148a-3p in predicting biochemical recurrence-was also validated by Zhao Z et al. [38]. Furthermore, the downregulated expression of miR-148a 3p has a high potential to predict lymphatic spread in locally advanced PCa [39].

Some studies have demonstrated the increased expression of miR-148a-3p in PCa, while contradictory findings have also been reported. Our analysis revealed that the expression of miR-148a-5p was downregulated in plasma samples compared to in BPH samples. Our study found that only 13.79% of patients had low-risk PCa and 41.37% had high-risk PCa. After the final histopathological examination, only 6.89% had a GS of 6. This finding could explain the decrease in miR-148a-3p expression observed in the PCa group compared to in the BPH group.

Despite inconsistent reports on the expression of miR-148a-3p in the literature, studies evaluating its biological role in PCa commonly indicate a tumor-suppressive effect. Sengupta et al. demonstrated that miR-148a-3p is downregulated in CRPC and identified DNA methyltransferase DNMT1. This gene is upregulated in various cancers and is considered an miR target [40]. Li G et al. illustrated the role of miR-148a-3p in PCa development. Methiltransferase-like 3 (METTL3) promoted by miR-146a-3p favors apoptosis and inhibits prostate tumor growth in nude mice [41]. MiR-146a-3p may target reticulon-4 (RTN4) in PCa tissues and cell lines. RTN4 may be phosphorylated by MAPKAPK2 and FYN at tyrosine 591 and serine 107, respectively. This suggested that RTN4 might somehow be involved in prostate tumor progression which opens up the potential for the creation or identification of selective agents targeting RTN4 for PCa therapy [42]. These studies prove that miR-148a-3p plays a role in the formation of a tumor-suppressive phenotype that inhibits cell survival, including PCa cells. This leads to it serving as both a reliable indicator of tumor progression and a possible biomarker for assessing the effectiveness of treatments in PCa.

Certain limits should be known when understanding the above findings. Firstly, the miRNA expression profiling was conducted on PCa patients versus BPH plasma; this might be additionally validated in urine samples. This might need to be additionally validated in urine samples. These transcripts will strengthen the robustness and clinical applicability of our results.

## 5. Conclusions

In conclusion, we identified a tumor-specific miRNA signature comprising two miRNAs. Combining these two transcripts has led to a slight improvement in overall sensitivity. This signature has the potential to serve as a novel minimally invasive biomarker for the diagnosis of PCa. Further validation of these miRNAs in larger patient cohorts and across different stages of PCa may strengthen their candidacy as clinically relevant biomarkers for PCa diagnosis and prognosis, particularly to recognize high-risk groups of PCa based on altered miRNA patterns. Additionally, investigating miRNAs’ biological functions and downstream targets could deliver insights into the molecular mechanisms underlying PCa pathogenesis.

## Figures and Tables

**Table 1:** MiRNA assays were used in the study.

No	miRNAs	Assay ID	Sequence
1	RNU48	001006	5′-GATGACCCCAGGTAACTCTGAGTGTGTCGCTGATGCCATCACCGCAGCGCTCTGACC-3’
2	U6	001973	5’-GTGCTCGCTTCGGCAGCACATATACTAAAATTGGAACGATACAGAGAAGATTAGCATGGCC CCTGCGCAAGGATGACACGCAAATTCGTGAAGCGTTCCATATTTT-3’
3	hsa-miR-106a-5p	000470	5’-AAAAGUGCUUACAGUGCAGGUAG-3’
4	hsa-miR-148a-3p	002169	5’-UCAGUGCACUACAGAACUUUGU-3’

**Table 2:** PCa patients’ characteristics and clinical and histopathological data.

Characteristics	PCa (n = 58)
Mean age (range), years	68.75 (58–75)
Mean PSA (range), ng/mL	11.05 (4.5–33)
EAU risk groups for biochemical recurrence of localized and locally advanced PCa, n (%)	
Low risk	8 (13.79%)
Intermediate risk	26 (44.82%)
High risk	24 (41.37%)
Pathological stage, n (%)	
T2	35 (60.34%)
T3a	9 (15.51%)
T3b	10 (17.24%)
T3a+b	1 (1.72%)
T4	3 (5.17%)
Pathological Gleason score prostatectomy, n (%)	
6	4 (6.89%)
7	42 (72.41%)
8	4 (6.89%)
9	8 (13.79%)
ISUP grade	
1	4 (6.89%)
2	23 (39.65%)
3	19 (32.75%)
4	4 (6.89%)
5	8 (13.79%)
Lymph node involvement, n (%)	3 (5.17)

EAU, European Association of Urology; PCa, prostate cancer; PSA, prostate-specific antigen; ISUP, International Society of Urological Pathology.

## Data Availability

The data that support the conclusion of this study will be made available by the authors on reasonable request.

## References

[1] Bergengren O., Pekala K.R., Matsoukas K., Fainberg J., Mungovan S.F., Bratt O., Bray F., Brawley O., Luckenbaugh A.N., Mucci L. (2023). 2022 Update on Prostate Cancer Epidemiology and Risk Factors-A Systematic Review. Eur. Urol..

[2] Merriel S.W.D., Pocock L., Gilbert E., Creavin S., Walter F.M., Spencer A., Hamilton W. (2022). Systematic review and meta-analysis of the diagnostic accuracy of prostate-specific antigen (PSA) for the detection of prostate cancer in symptomatic patients. BMC Med..

[3] Carvalhal G.F., Smith D.S., Mager D.E., Ramos C., Catalona W.J. (1999). Digital rectal examination for detecting prostate cancer at prostate specific antigen levels of 4 ng/mL or less. J. Urol..

[4] Gosselaar C., Roobol M.J., Roemeling S., Schröder F.H. (2008). The role of the digital rectal examination in subsequent screening visits in the European randomized study of screening for prostate cancer (ERSPC), Rotterdam. Eur. Urol..

[5] Oerther B., Engel H., Bamberg F., Sigle A., Gratzke C., Benndorf M. (2022). Cancer detection rates of the PI-RADSv2.1 assessment categories: Systematic review and meta-analysis on lesion level and patient level. Prostate Cancer Prostatic Dis..

[6] Yilmaz M., Toprak T., Suarez-Ibarrola R., Sigle A., Gratzke C., Miernik A. (2022). Incidental prostate cancer after holmium laser enucleation of the prostate-A narrative review. Andrologia.

[7] Matanhelia D.M., Croghan S., Nason G.J., O’Connell C., Galvin D.J. (2019). The Management of Incidental Prostate Cancer Following TURP. Ir. Med. J..

[8] Esquela-Kerscher A., Slack F.J. (2006). Oncomirs—microRNAs with a role in cancer. Nat. Rev. Cancer.

[9] Bartel D.P. (2018). Metazoan MicroRNAs. Cell.

[10] Koppers-Lalic D., Hackenberg M., de Menezes R., Misovic B., Wachalska M., Geldof A., Zini N., de Reijke T., Wurdinger T., Vis A. (2016). Non-invasive prostate cancer detection by measuring miRNA variants (isomiRs) in urine extracellular vesicles. Oncotarget.

[11] Mitchell P.S., Parkin R.K., Kroh E.M., Fritz B.R., Wyman S.K., Pogosova-Agadjanyan E.L., Peterson A., Noteboom J., O’Briant K.C., Allen A. (2008). Circulating microRNAs as stable blood-based markers for cancer detection. Proc. Natl. Acad. Sci. USA.

[12] Khan M.M., Serajuddin M., Bharadwaj M. (2023). Potential plasma microRNAs signature miR-190b-5p, miR-215-5p and miR-527 as non-invasive biomarkers for prostate cancer. Biomarkers.

[13] Joković S.M., Dobrijević Z., Kotarac N., Filipović L., Popović M., Korać A., Vuković I., Savić-Pavićević D., Brajušković G. (2022). MiR-375 and miR-21 as Potential Biomarkers of Prostate Cancer: Comparison of Matching Samples of Plasma and Exosomes. Genes.

[14] Turchinovich A., Weiz L., Burwinkel B. (2012). Extracellular miRNAs: The mystery of their origin and function. Trends Biochem. Sci..

[15] Cochetti G., Poli G., Guelfi G., Boni A., Egidi M.G., Mearini E. (2016). Different levels of serum microRNAs in prostate cancer and benign prostatic hyperplasia: Evaluation of potential diagnostic and prognostic role. OncoTargets Ther..

[16] Yang G., Li T., Liu J., Quan Z., Liu M., Guo Y., Wu Y., Ou L., Wu X., Zheng Y. (2023). lncRNA MAGI2-AS3 suppresses castration-resistant prostate cancer proliferation and migration via the miR-106a-5p/RAB31 axis. Genomics.

[17] Dybos S.A., Flatberg A., Halgunset J., Viset T., Rolfseng T., Kvam S., Skogseth H. (2018). Increased levels of serum miR-148a-3p are associated with prostate cancer. Apmis.

[18] Walter B.A., Valera V.A., Pinto P.A., Merino M.J. (2013). Comprehensive microRNA profiling of prostate cancer. J. Cancer.

[19] Rao X., Huang X., Zhou Z., Lin X. (2013). An improvement of the 2^(–delta delta CT) method for quantitative real-time polymerase chain reaction data analysis. Biostat. Bioinforma Biomath..

[20] Vlachos I.S., Zagganas K., Paraskevopoulou M.D., Georgakilas G., Karagkouni D., Vergoulis T., Dalamagas T., Hatzigeorgiou A.G. (2015). DIANA-miRPath v3.0: Deciphering microRNA function with experimental support. Nucleic Acids Res..

[21] Licursi V., Conte F., Fiscon G., Paci P. (2019). MIENTURNET: An interactive web tool for microRNA-target enrichment and network-based analysis. BMC Bioinform..

[22] http://combiroc.eu.

[23] http://userver.bio.uniroma1.it/apps/mienturnet/#tab-2530-2.

[24] Saini S. (2016). PSA and beyond: Alternative prostate cancer biomarkers. Cell. Oncol..

[25] Schitcu V.H., Raduly L., Nutu A., Zanoaga O., Ciocan C., Munteanu V.C., Cojocneanu R., Petrut B., Coman I., Braicu C. (2022). MicroRNA Dysregulation in Prostate Cancer. Pharmacogenomics Pers. Med..

[26] Schitcu V.H., Raduly L., Zanoaga O., Jurj A., Munteanu V.C., Budisan L., Petrut B., Braicu C., Coman I., Berindan-Neagoe I. (2023). TP53 gene implications in prostate cancer evolution: Potential role in tumor classification. Med. Pharm. Rep..

[27] Zhao H., Tian X., He L., Li Y., Pu W., Liu Q., Tang J., Wu J., Cheng X., Liu Y. (2018). Apj+ Vessels Drive Tumor Growth and Represent a Tractable Therapeutic Target. Cell Rep..

[28] Lin T.H., Chang S.L., Khanh P.M., Trang N.T.N., Liu S.C., Tsai H.C., Chang A.-C., Lin J.-Y., Chen P.-C., Liu J.-F. (2022). Apelin Promotes Prostate Cancer Metastasis by Downregulating TIMP2 via Increases in miR-106a-5p Expression. Cells.

[29] Dhar S., Kumar A., Rimando A.M., Zhang X., Levenson A.S. (2015). Resveratrol and pterostilbene epigenetically restore PTEN expression by targeting oncomiRs of the miR-17 family in prostate cancer. Oncotarget.

[30] Li Y., Deng X., Zeng X., Peng X. (2016). The Role of Mir-148a in Cancer. J. Cancer.

[31] Paunescu I.A., Bardan R., Marcu A., Nitusca D., Dema A., Negru S., Balacescu O., Balacescu L., Cumpanas A., Sirbu I.O. (2019). Biomarker Potential of Plasma MicroRNA-150-5p in Prostate Cancer. Medicina.

[32] He W., Zhang F., Jiang F., Liu H., Wang G. (2022). Correlations between serum levels of microRNA-148a-3p and microRNA-485-5p and the progression and recurrence of prostate cancer. BMC Urol..

[33] Stuopelyte K., Daniunaite K., Bakavicius A., Lazutka J.R., Jankevicius F., Jarmalaite S. (2016). The utility of urine-circulating miRNAs for detection of prostate cancer. Br. J. Cancer.

[34] Szczyrba J., Löprich E., Wach S., Jung V., Unteregger G., Barth S., Grobholz R., Wieland W., Stöhr R., Hartmann A. (2010). The MicroRNA profile of prostate carcinoma obtained by deep sequencing. Mol. Cancer Res..

[35] Fujita Y., Kojima K., Ohhashi R., Hamada N., Nozawa Y., Kitamoto A., Sato A., Kondo S., Kojima T., Deguchi T. (2010). MiR-148a attenuates paclitaxel resistance of hormone-refractory, drug-resistant prostate cancer PC3 cells by regulating MSK1 expression. J. Biol. Chem..

[36] Lichner Z., Ding Q., Samaan S., Saleh C., Nasser A., Al-Haddad S., Samuel J.N., E Fleshner N., Stephan C., Jung K. (2015). miRNAs dysregulated in association with Gleason grade regulate extracellular matrix, cytoskeleton and androgen receptor pathways. J. Pathol..

[37] Porkka K.P., Pfeiffer M.J., Waltering K.K., Vessella R.L., Tammela T.L.J., Visakorpi T. (2007). MicroRNA Expression profiling in prostate cancer. Cancer Res..

[38] Zhao Z., Weickmann S., Jung M., Lein M., Kilic E., Stephan C., Erbersdobler A., Fendler A., Jung K. (2019). A Novel Predictor Tool of Biochemical Recurrence after Radical Prostatectomy Based on a Five-MicroRNA Tissue Signature. Cancers.

[39] Pudova E.A., Kobelyatskaya A.A., Katunina I.V., Snezhkina A.V., Fedorova M.S., Pavlov V.S., Bakhtogarimov I.R., Lantsova M.S., Kokin S.P., Nyushko K.M. (2023). Lymphatic Dissemination in Prostate Cancer: Features of the Transcriptomic Profile and Prognostic Models. Int. J. Mol. Sci..

[40] Sengupta D., Deb M., Patra S.K. (2018). Antagonistic activities of miR148a and DNMT1: Ectopic expression of miR-148a impairs DNMT1 mRNA and dwindle cell proliferation and survival. Gene.

[41] Li G., Liu J., Wang Y., Liu H., Fu J., Zhao Y., Huang Y. (2023). METTL3-mediated m6A modification of pri-miR-148a-3p affects prostate cancer progression by regulating TXNIP. Environ. Toxicol..

[42] Zhao H., Su W., Zhu C., Zeng T., Yang S., Wu W., Wang D. (2019). Cell fate regulation by reticulon-4 in human prostate cancers. J. Cell. Physiol..

